# Noninvasive, simultaneous, and continuous measurements of stroke volume and tidal volume using EIT: feasibility study of animal experiments

**DOI:** 10.1038/s41598-020-68139-3

**Published:** 2020-07-09

**Authors:** Geuk Young Jang, You Jeong Jeong, Tingting Zhang, Tong In Oh, Ryoung-Eun Ko, Chi Ryang Chung, Gee Young Suh, Eung Je Woo

**Affiliations:** 1 Department of Biomedical Engineering, College of Medicine, Kyung Hee University, Seoul, 02447 Korea; 2 Department of Medical Engineering, Graduate School, Kyung Hee University, Seoul, 02447 Korea; 3 Department of Critical Care Medicine, Samsung Medical Center, Sungkyunkwan University School of Medicine, Seoul, 06351 Korea

**Keywords:** Biomedical engineering, Preclinical research

## Abstract

Currently, there is no noninvasive method available for simultaneous measurements of tidal volume and stroke volume. Electrical impedance tomography (EIT) has been used for regional lung ventilation imaging. Cardiac EIT imaging, however, has not been successful due to the technical difficulty in extracting weak cardiogenic components. Instead of regional imaging, in this paper, we use the EIT technique to simultaneously measure two global variables of tidal volume and stroke volume. Time-varying patterns of boundary voltage data originating from lung ventilation and cardiac blood flow were extracted from measured boundary voltage data using the principal component analysis (PCA) and independent component analysis (ICA). The source consistency theory was adopted to separately synthesize time-series of boundary voltage data associated with lung ventilation and cardiac blood flow. The respiratory volume signal (RVS) and cardiac volume signal (CVS) were extracted from reconstructed time-difference EIT images of lung ventilation and cardiac blood flow, respectively. After calibrating the volume signals using the mechanical ventilator and the invasive transpulmonary thermodilution (TPTD) method, tidal volume and stroke volume were computed as valley-to-peak values of the RVS and CVS, respectively. The difference in the tidal volume data between EIT and mechanical ventilator was within ± 20 ml from six pigs. The difference in the stroke volume data between EIT and TPTD was within ± 4.7 ml from the same animals. The results show the feasibility of the proposed method as a new noninvasive cardiopulmonary monitoring tool for simultaneous continuous measurements of stroke volume and tidal volume that are two most important vital signs.

## Introduction

Stroke volume and cardiac output are key indicators of hemodynamically-unstable patients in operating room (OR), intensive care unit (ICU), and emergency room (ER). Hypoventilation could be detected by continuous monitoring of tidal volume and minute ventilation from non-intubated patients with respiratory depression in post anesthesia care unit (PACU) and general ward. Simultaneous measurements of stroke volume and tidal volume will enable monitoring of cardiopulmonary functions in an integrated way. Noninvasive, simultaneous, and continuous measurements of stroke volume and tidal volume, therefore, would benefit many critically-ill patients in OR, ICU, PACU, ER, and other places in hospitals.

Electrical impedance tomography (EIT) has been studied for image-based noninvasive monitoring of physiological functions such as lung ventilation and cardiac blood flow^1–4^. The image contrast in EIT is the electrical tissue conductivity^5^, which is probed by injecting AC currents and measuring boundary voltages from multiple electrodes attached around the chest. A linearized image reconstruction algorithm using a sensitivity matrix derived from the lead field theory can be adopted to produce a time series of time-difference EIT images of internal physiological functions^4,6,7^.

EIT has been mainly used for regional lung ventilation imaging during mechanical ventilation for lung protective ventilation^8,9^. However, cardiac EIT imaging has not been successful yet primarily due to the following reasons. First, lung ventilation, cardiac blood flow, movements of internal organs, and chest motions occur simultaneously and measured boundary voltage data are influenced by all of them. Second, the influence of cardiac blood flow on the measured boundary voltage data is much weaker than that of lung ventilation. Third, time-varying boundary voltage data associated with lung ventilation and cardiac blood flow have overlapping frequency spectra even though their fundamental frequencies could be different. Fourth, an EIT system with high temporal resolution is needed to capture fast changes in cardiac blood flow.

Numerous techniques have been developed to separate lung ventilation and cardiac blood flow components in EIT using breath holding, ECG-triggered signal averaging, bandpass filtering, and parametric methods^10–12^. Recently, the principal component analysis (PCA) has been adopted to extract time-varying cardiac blood flow components from pixels or regions-of-interest (ROIs) in reconstructed time-difference EIT images^13,14^. The independent component analysis (ICA) was also suggested to extract cardiac and respiratory signals from reconstructed EIT images^15^. However, since the sources of cardiogenic changes in EIT images are not well understood, interpretation of cardiac EIT images is still controversial^16–18^. Though previous studies showed the feasibility of cardiac EIT imaging, more technical improvements are needed in terms of accuracy and reliability for imaging regional changes^19–21^.

In this paper, instead of regional imaging, we focus on simultaneously measuring two global variables of tidal volume and stroke volume using EIT. The PCA and ICA are used to separately extract shape-reference voltage waveforms corresponding to lung ventilation and cardiac blood flow. Unlike the previous studies of applying the PCA or ICA to reconstructed EIT images, we apply both methods to measured boundary voltage data before EIT image reconstructions. Adopting the lately developed source consistency EIT method^22^, boundary voltage data corresponding to lung ventilation and cardiac blood flow are synthesized from the extracted shape-reference voltage waveforms. These novel preprocessing methods are implemented in an EIT system with 100 frames/s temporal resolution to separately and simultaneously extract respiratory volume and cardiac volume signals from reconstructed time-difference EIT images using the synthesized data.

We will briefly describe the adopted EIT system emphasizing its fast temporal resolution. After explaining the PCA and ICA methods to extract shape-reference waveforms of lung ventilation and cardiac blood flow components, the source consistency method to synthesize boundary voltage data will be described. The proposed methods will be applied to animal experiments using six pigs for performance validation.

## Methods

### EIT system and data collection

The adopted EIT device was a KHU Mark 2.5 sixteen-channel EIT system^23–27^. A pipeline structure of sequential current injections and voltage measurements was implemented to produce one EIT image in 10 ms. A resistor phantom was included in the device for calibrations and performance evaluation. The maximum signal-to-noise ratio (SNR) of the voltage measurements was about 80 dB.

Currents were injected between a chosen adjacent electrode pair and boundary voltage data were measured between all sixteen adjacent electrode pairs. Sequentially injecting currents between all sixteen adjacent electrode pairs, $16\times 16=256$ voltage data could be acquired to comprise one scan. For each current injection, however, three voltage data from three adjacent electrode pairs including the current-injection electrodes were discarded since they were affected by the contact impedances of the current-injection electrodes. From consecutive scans at 100 frames/s for *T* s, $16\times (16-3)=208$ time series of measured boundary voltage data were obtained. The length of each time series is denoted as $N=100\times T$. Each one of these time-series is called a voltage channel in this paper. Note that each voltage channel is influenced by the following factors: lung ventilation, cardiac blood flow, movements of internal organs, chest motions of respiratory efforts, body position changes, measurement noise and interferences, boundary geometry of the chest, and electrode positions, shapes, and sizes.

In time-difference EIT, errors in boundary geometry and electrode configurations are minimized using the difference between a set of voltage data at certain time and another set at a chosen reference time. In this paper, since we separately extract voltage channel data corresponding to lung ventilation and cardiac blood flow, subsequent time-difference EIT image reconstructions will be also done separately.

### Extraction of shape-reference waveforms

We denote the $N\times 208$ data matrix $\mathbf{X}$ of all 208 voltage channels as

$$\begin{aligned} \mathbf{X}=\left( \begin{array}{cccc} \uparrow &{} \uparrow &{} \cdots &{} \uparrow \\ \mathbf{x}_1 &{} \mathbf{x}_2 &{} \cdots &{} \mathbf{x}_{208} \\ \downarrow &{} \downarrow &{} \cdots &{} \downarrow \\ \end{array} \right) \end{aligned}$$

where $\mathbf{x}_i$ for $i=1,2,\ldots ,208$ is the $N\times 1$ data vector of the *i*th voltage channel. We apply the PCA to $\mathbf{X}$ along the time axis and extract the first principal component as the shape-reference waveform of lung ventilation denoted as $\mathbf{l}$. The choice of the first principal component was based on the observation that lung ventilation produces largest changes in measured boundary voltage data in the absence excessive motion artifacts. The PCA was implemented using the singular value decomposition (SVD) of $\mathbf{X}\mathbf{X}^T$ as

1 $$\begin{aligned} \mathbf{X}\mathbf{X}^T=\sum _{j=1}^{N}\lambda _j\mathbf{u}_j\mathbf{u}_j^T \end{aligned}$$

where $\lambda _j$ for $j=1,2,\ldots ,N$ are the singular values in descending order and $\mathbf{u}_j$ are the corresponding singular vectors. We set the shape-reference waveform of lung ventilation as

2 $$\begin{aligned} \mathbf{l}=\mathbf{u}_1. \end{aligned}$$

We form the following matrix ${\hat{\mathbf{U}}}$ as

3 $$\begin{aligned} {\hat{\mathbf{U}}}=\left( \begin{array}{cccc} \uparrow &{} \uparrow &{} \cdots &{} \uparrow \\ \mathbf{u}_2 &{} \mathbf{u}_3 &{} \cdots &{} \mathbf{u}_M \\ \downarrow &{} \downarrow &{} \cdots &{} \downarrow \\ \end{array} \right) \end{aligned}$$

which is an $N\times (M-1)$ matrix where $M<208$. Note that we excluded $\mathbf{u}_1$ to suppress the influence of lung ventilation as much as possible in the following step. Applying the ICA to ${\hat{\mathbf{U}}}^T$, we get

4 $$\begin{aligned} \mathbf{S}^T=\mathbf{W}{\hat{\mathbf{U}}}^T \end{aligned}$$

where $\mathbf{W}$ is an $(M-1)\times (M-1)$ unmixing matrix and $\mathbf{S}$ is an $N\times (M-1)$ source signal matrix expressed as

5 $$\begin{aligned} \mathbf{S}=\left( \begin{array}{cccc} \uparrow &{} \uparrow &{} \cdots &{} \uparrow \\ \mathbf{s}_1 &{} \mathbf{s}_2 &{} \cdots &{} \mathbf{s}_{M-1} \\ \downarrow &{} \downarrow &{} \cdots &{} \downarrow \\ \end{array} \right) . \end{aligned}$$

To extract the shape-reference waveform of cardiac blood flow, we apply the fast Fourier transform to $\mathbf{s}_m$ for $m=1,2,\ldots ,(M-1)$ and obtain their frequency spectra $\Psi (\mathbf{s}_m)$. Among $(M-1)$ frequency spectra, we choose one source signal $\mathbf{s}_h$ with the largest energy at the fundamental frequency $f_h$ of the heart rate. The shape-reference waveform of cardiac blood flow $\mathbf{h}$ is

6 $$\begin{aligned} \mathbf{h}=\mathbf{s}_h. \end{aligned}$$

**Figure 1:**
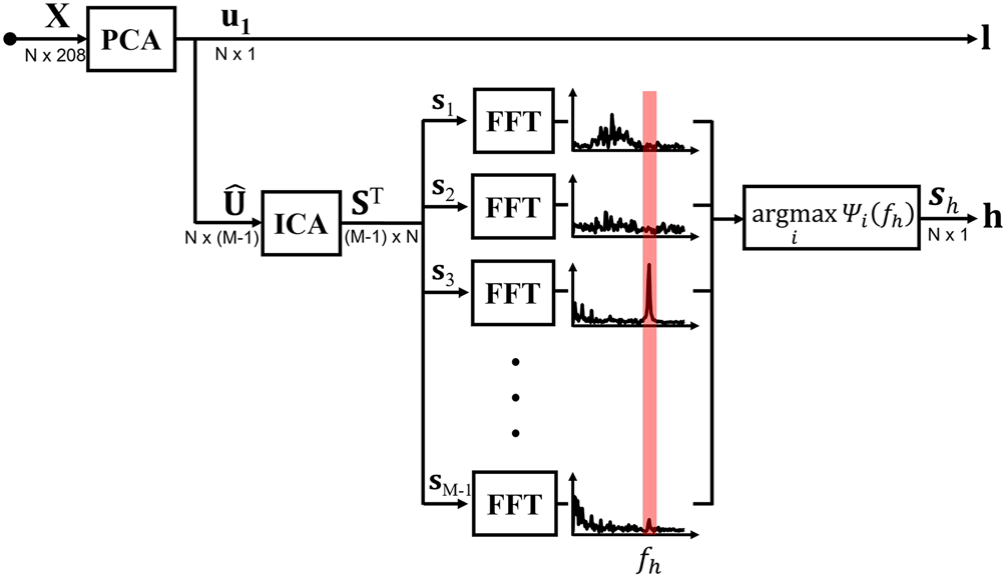
Block diagram of the data separation method. The PCA and ICA techniques are adopted to extract shape-reference voltage waveforms $\mathbf{l}$ and $\mathbf{h}$ corresponding to lung ventilation and cardiac blood flow, respectively.

Figure 1 shows the block diagram of the process to separately extract shape-reference waveforms of $\mathbf{l}$ and $\mathbf{h}$.

### Syntheses of voltage channel data

When there exists a single time-varying conductivity source or physiological function inside the chest, the recent source consistency method showed that the following observations hold^22^.

- The shapes of all time-varying voltage channels are identical up to scaling constants and offsets.
- The shape of each time-varying voltage channel is determined by the shape of the time-varying conductivity waveform of the source.

For multiple time-varying conductivity sources, each voltage channel influenced by all sources can be approximated as a weighted sum of shape-reference voltage waveforms of the individual sources.

Using the source consistency theory, 208 voltage channels $\mathbf{x}_{l,i}$ for $i=1,2,\ldots ,208$ associated with lung ventilation are expressed as

7 $$\begin{aligned} \mathbf{x}_{l,i}=a_i\mathbf{l}+ d_i \end{aligned}$$

where $a_i$ and $d_i$ are the scale factor and offset of the *i*th voltage channel associated with lung ventilation, respectively. Similarly, 208 voltage channels $\mathbf{x}_{h,i}$ for $i=1,2,\ldots ,208$ associated with cardiac blood flow are expressed as

8 $$\begin{aligned} \mathbf{x}_{h,i}=b_i\mathbf{h}+ e_i \end{aligned}$$

where $b_i$ and $e_i$ are the scale factor and offset of the *i*th voltage channel associated with cardiac blood flow, respectively.

In the absence of excessive motion artifacts, we set the sum of $\mathbf{x}_{l,i}$ and $\mathbf{x}_{h,i}$ to be approximately equal to the *i*th voltage channel data $\mathbf{x}_i$ as follows:

9 $$\begin{aligned} \mathbf{x}_{l,i}+\mathbf{x}_{h,i}=a_i\mathbf{l}+ b_i\mathbf{h}+ c_i \approx \mathbf{x}_i \end{aligned}$$

where $c_i=d_i+e_i$. Using the least square method, we can compute the matrix $\mathbf{C}$ of the scale factors and offsets for all 208 channels as

10 $$\begin{aligned} \mathbf{C}=\left( \mathbf{B}^T\mathbf{B}\right) ^{-1}\mathbf{B}^T\mathbf{X}\end{aligned}$$

where

$$\begin{aligned} \mathbf{C}=\left( \begin{array}{cccc} a_1 &{} a_2 &{} \cdots &{} a_{208} \\ b_1 &{} b_2 &{} \cdots &{} b_{208} \\ c_1 &{} c_2 &{} \cdots &{} c_{208} \\ \end{array} \right) \end{aligned}$$

and

$$\begin{aligned} \mathbf{B}^T=\left( \begin{array}{ccc} \longleftarrow &{} \mathbf{h}^T &{} \longrightarrow \\ \longleftarrow &{} \mathbf{l}^T &{} \longrightarrow \\ 1 &{} \cdots &{} 1 \\ \end{array} \right) . \end{aligned}$$

Using the scale factors and offsets in $\mathbf{C}$, all $\mathbf{x}_{l,i}$ and $\mathbf{x}_{h,i}$ for $i=1,2,\ldots ,208$ are computed to synthesize two $N\times 208$ data matrices $\mathbf{X}_l$ and $\mathbf{X}_h$ as

$$\begin{aligned} \mathbf{X}_l=\left( \begin{array}{cccc} \uparrow &{} \uparrow &{} \cdots &{} \uparrow \\ \mathbf{x}_{l,1} &{} \mathbf{x}_{l,2} &{} \cdots &{} \mathbf{x}_{l,208} \\ \downarrow &{} \downarrow &{} \cdots &{} \downarrow \\ \end{array} \right) \end{aligned}$$

and

$$\begin{aligned} \mathbf{X}_h=\left( \begin{array}{cccc} \uparrow &{} \uparrow &{} \cdots &{} \uparrow \\ \mathbf{x}_{h,1} &{} \mathbf{x}_{h,2} &{} \cdots &{} \mathbf{x}_{h,208} \\ \downarrow &{} \downarrow &{} \cdots &{} \downarrow \\ \end{array} \right) \end{aligned}$$

corresponding to lung ventilation and cardiac blood flow, respectively. Note that we can set $d_i=e_i=0.5c_i$ since the offsets of $d_i$ and $e_i$ are irrelevant in time-difference EIT image reconstructions where voltage differences are used.

### Image reconstructions and volume signals

The fidelity-embedded regularization (FER) algorithm was adopted for time-difference EIT image reconstructions^28^. To incorporate the subject-specific boundary shape into the sensitivity matrix computation, a 3D Scanner (Sense, 3D Systems, U.S.A.) was used to capture the boundary shape of each pig. Using the synthesized data matrices $\mathbf{X}_l$ and $\mathbf{X}_h$, two time-series of time-difference EIT images $\mathbf{I}_l$ and $\mathbf{I}_h$ corresponding to lung ventilation and cardiac blood flow, respectively, were reconstructed. From the reconstructed time-difference images, we defined ROIs where the respiratory volume signal (RVS) and cardiac volume signal (CVS) were extracted as sums of pixel values within the ROIs. All computations were done using MATLAB (The MathWorks, U.S.A.).

### Animal experiments

**Figure 2:**
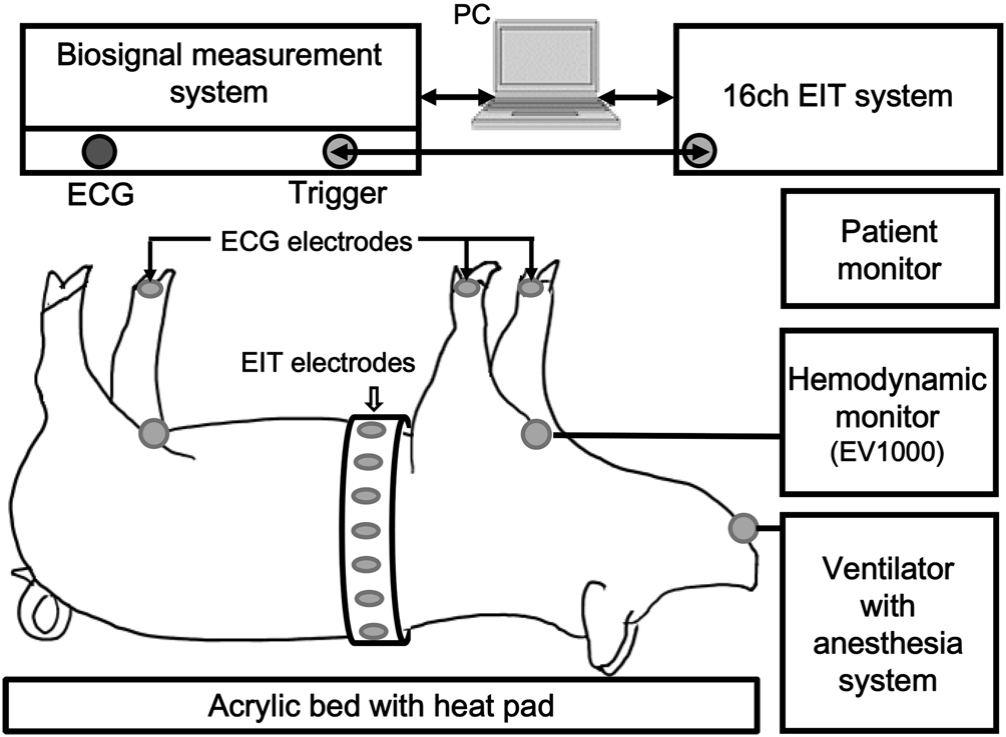
Experimental setup for in vivo chest EIT imaging.

The animal experiment was approved by the Institutional Animal Care and Use Committee (SMC-20150804001, Samsung Medical Center, Seoul, Korea). All experiments using six normal pigs (average weight of 29± 2 kg and age of 6 month) were performed in accordance with relevant guidelines and regulations. Figure 2 shows the setup for the animal experiments. Each animal was premedicated with intramuscular injection of ketamine (20 mg/kg) and xylazine (2.5 mg/kg). The animal was connected to a mechanical ventilator (Hamilton-G5, Hamilton Medical, Switzerland) by tracheal intubation. Anesthesia was maintained by inhalation of 2% isoflurane mixed with 25% oxygen. Throughout the experiment, vital signs were monitored using a patient monitor (IntelliVue MP50, Philips, Netherlands). The respiration rate was 20 breaths/min and the heart rate was in the range of 70–100 beats/min. The supplied air volume from the ventilator was 8 ml/kg at the beginning of the experiment and increased to 18 ml/kg in four steps. Cardiac output and stroke volume were independently measured using an invasive hemodynamic monitor (EV1000, Edwards Lifesciences, USA) in the transpulmonary thermodilution (TPTD) mode.

## Results

**Figure 3:**
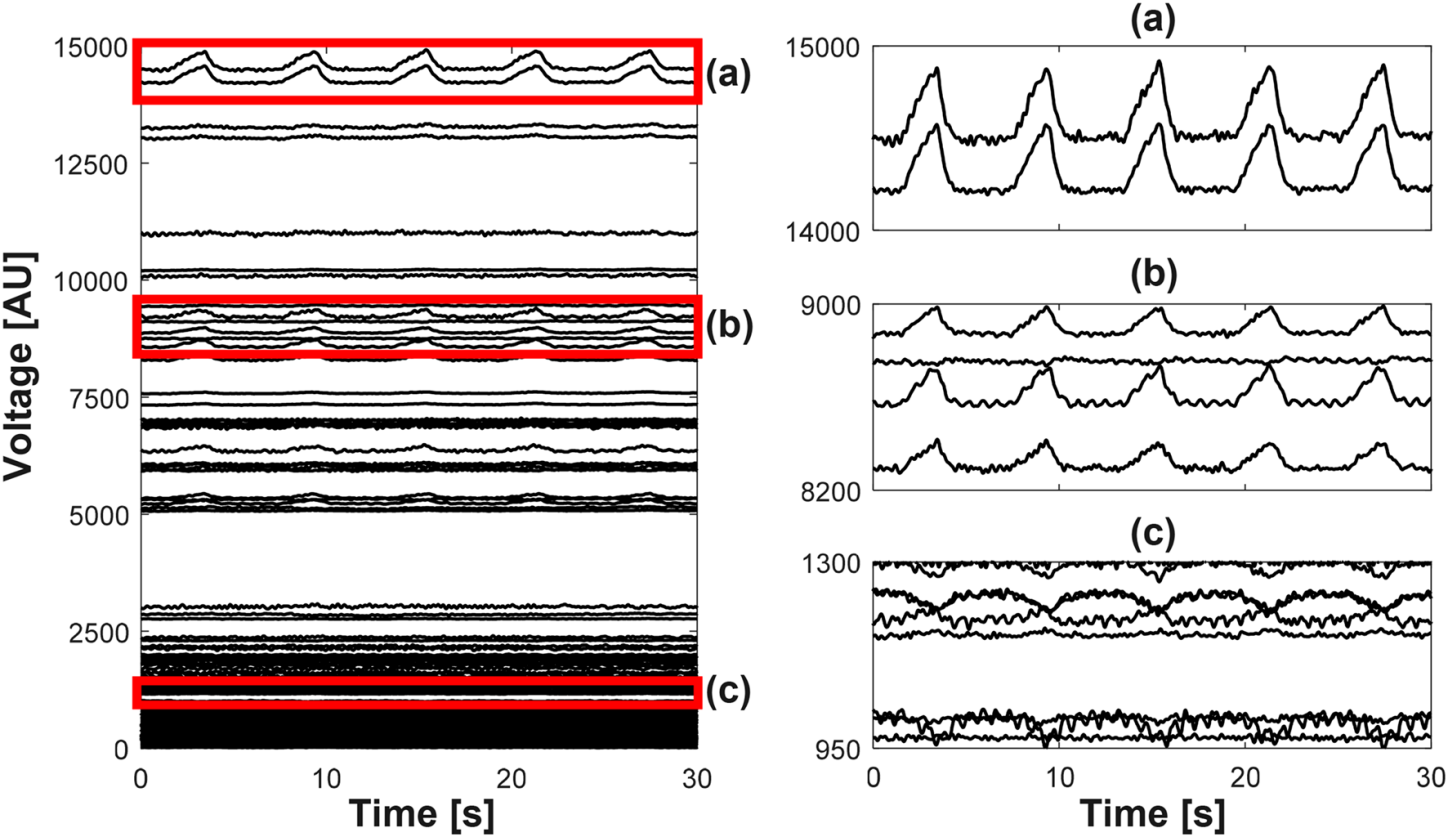
Measured data $\mathbf{X}$ of 208 voltage channels from a mechanically ventilated pig. (**a**–**c**) are enlarged views of several channels.

**Figure 4:**
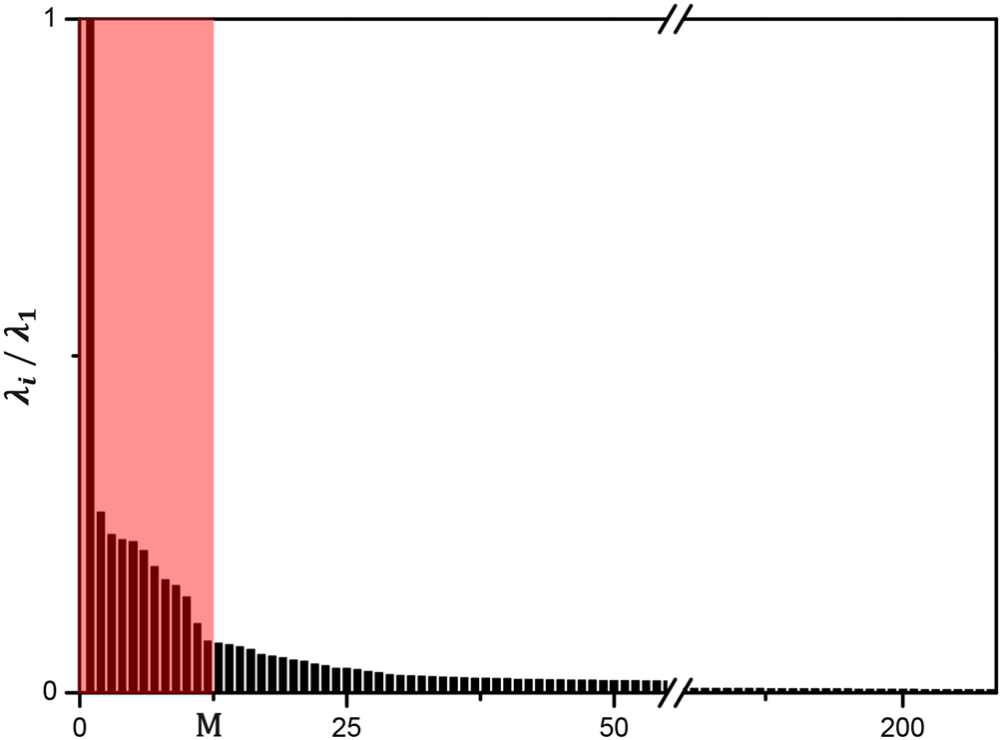
Normalized singular values $\frac{\lambda _i}{\lambda _1}$ for $i=1,2,\ldots ,208$. Twelve singular vectors corresponding to the largest twelve singular values were chosen to approximate the voltage data.

Figure 3 plots measured 208 voltage channels from the first animal during mechanical ventilation. Figure 3a–c are enlarged views of several voltage channels. Figure 4 shows the distribution of the normalized singular values $\frac{\lambda _i}{\lambda _1}$ for $i=1,2,\ldots ,208$ after applying the PCA to the voltage data in Fig. 3. The first principal component was selected as the shape-reference waveform for lung ventilation denoted as $\mathbf{l}$.

**Figure 5:**
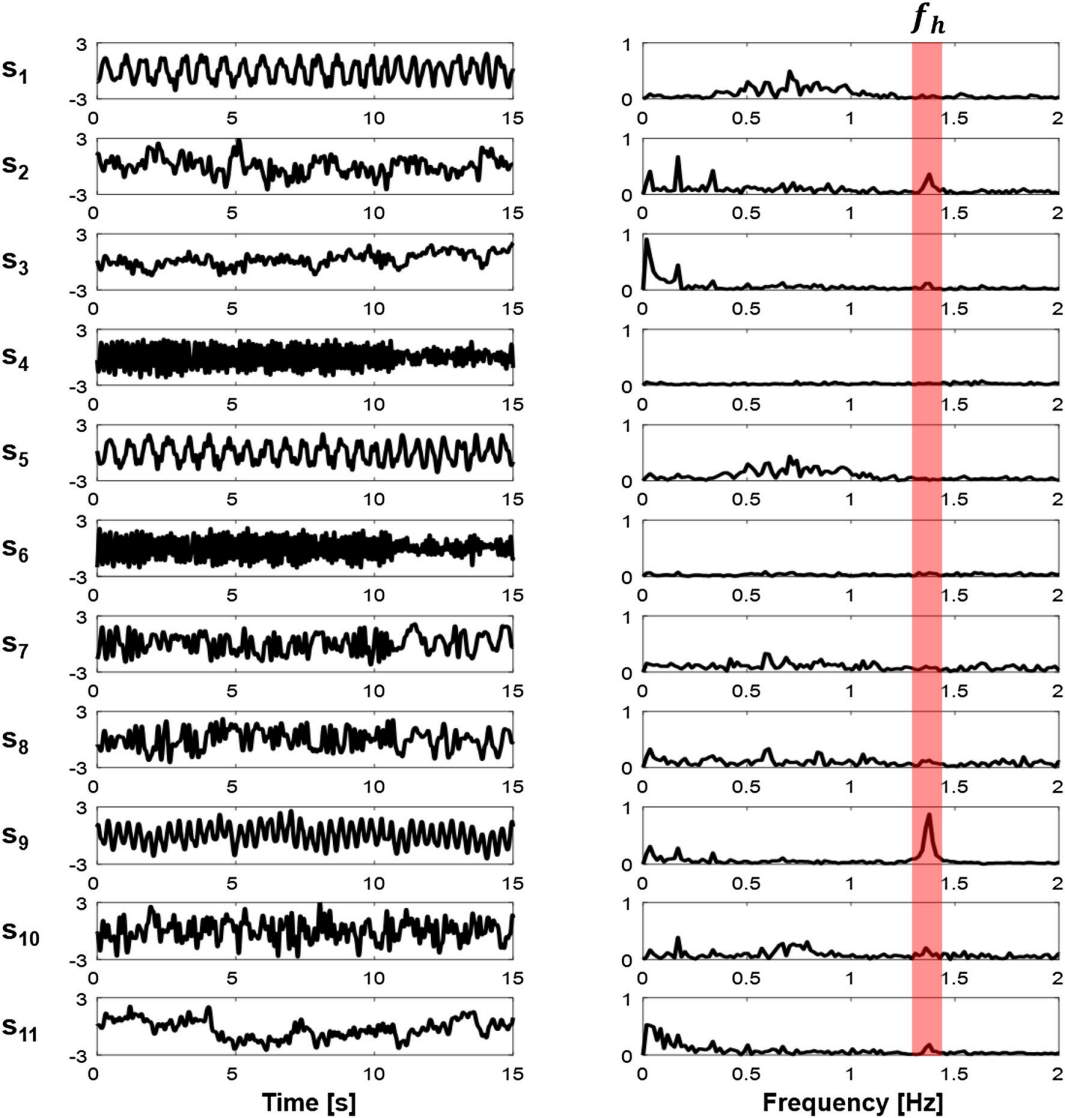
Separated independent components and their frequency spectra. The fundamental frequency of cardiac blood flow is denoted as $f_h$.

**Figure 6:**
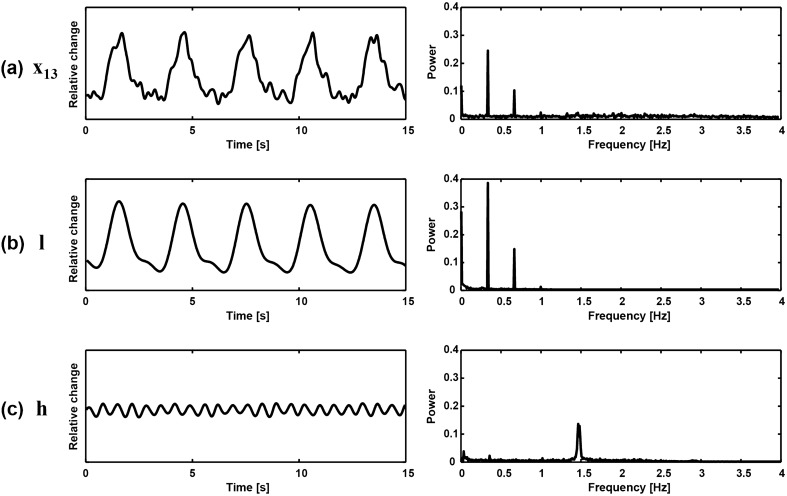
(**a**) Typical voltage channel data and its spectrum including both lung ventilation and cardiac blood flow components. (**b**) Separated lung ventilation component $\mathbf{l}$ and its spectrum. (**c**) Separated cardiac blood flow component $\mathbf{h}$ and its spectrum.

Using the ICA applied to the principal components of $\mathbf{u}_2,\mathbf{u}_3,\ldots ,\mathbf{u}_{12}$ excluding $\mathbf{u}_1$, we computed the source signals and their frequency spectra as shown in Fig. 5. In this example, we selected the ninth independent component $\mathbf{s}_9$ as the shape-reference waveform $\mathbf{h}$ of cardiac blood flow. Figure 6a shows a typical voltage channel with its power spectrum while (b) and (c) plot the extracted shape-reference waveforms $\mathbf{l}$ and $\mathbf{h}$ and their power spectra, respectively.

**Figure 7:**
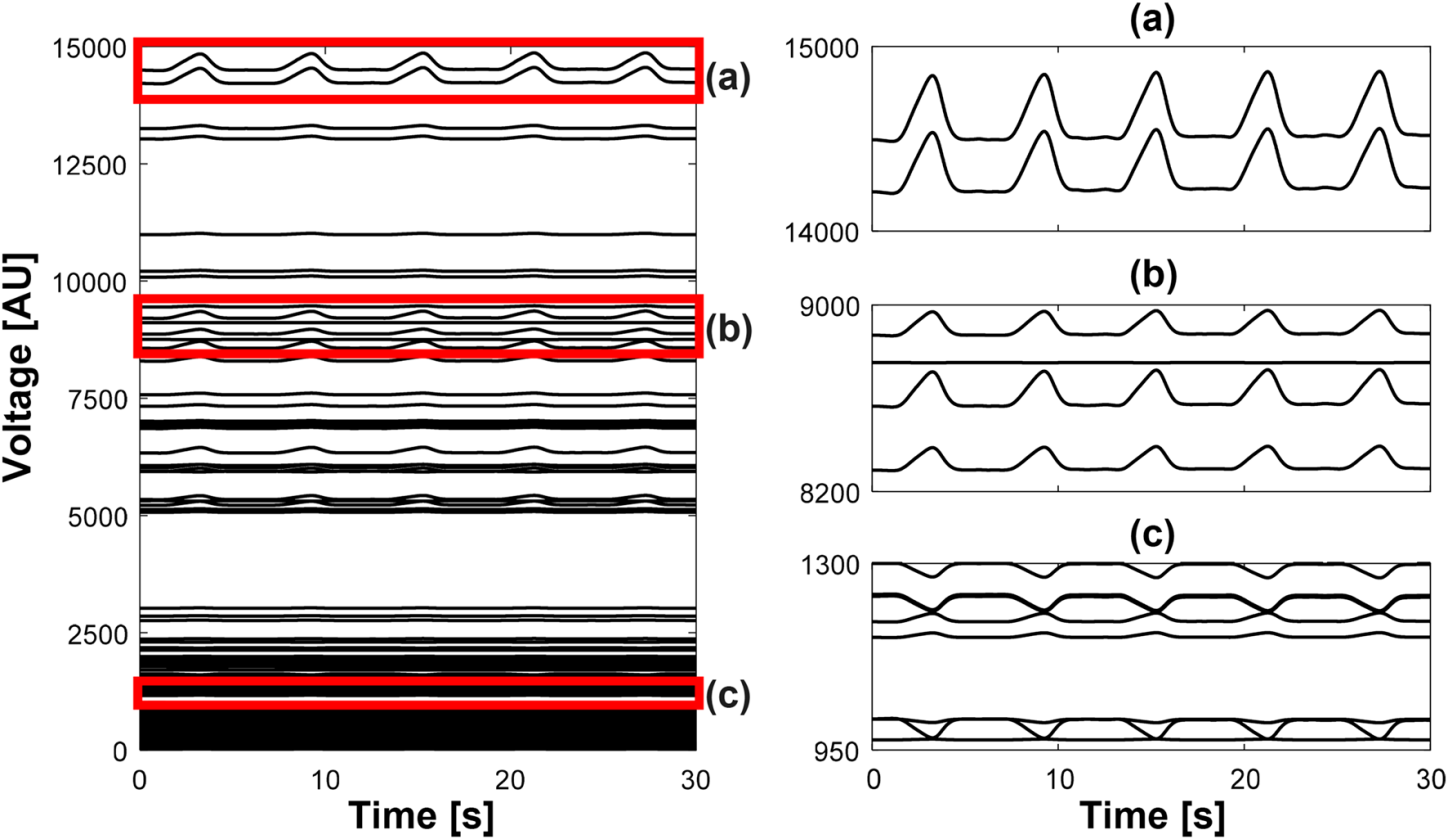
Synthesized voltage data $\mathbf{X}_l$ of lung ventilation. (**a**–**c** are enlarged views of several channels.

**Figure 8:**
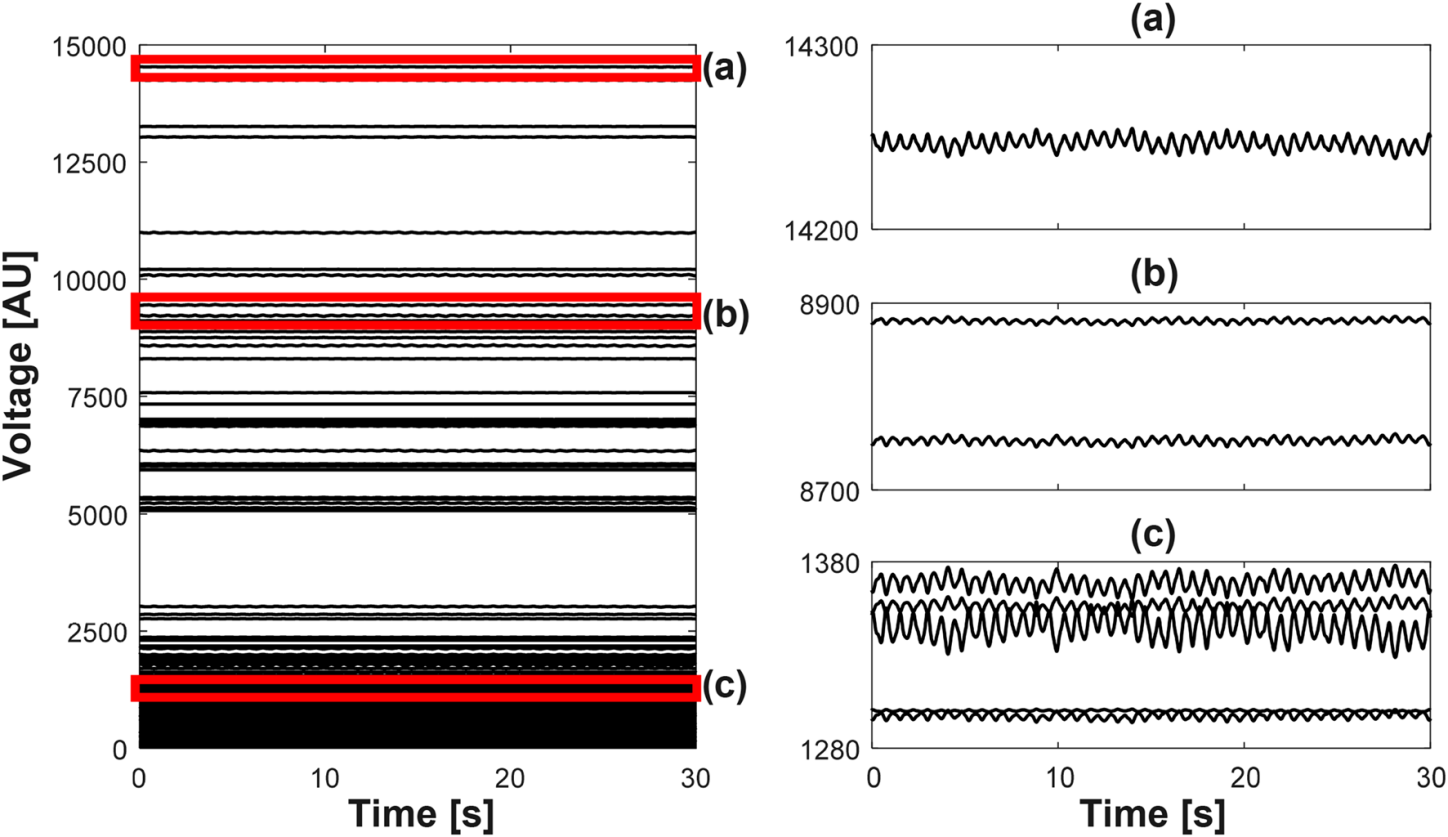
Synthesized voltage data $\mathbf{X}_h$ of cardiac blood flow. (**a**–**c**) are enlarged views of several channels.

**Figure 9:**
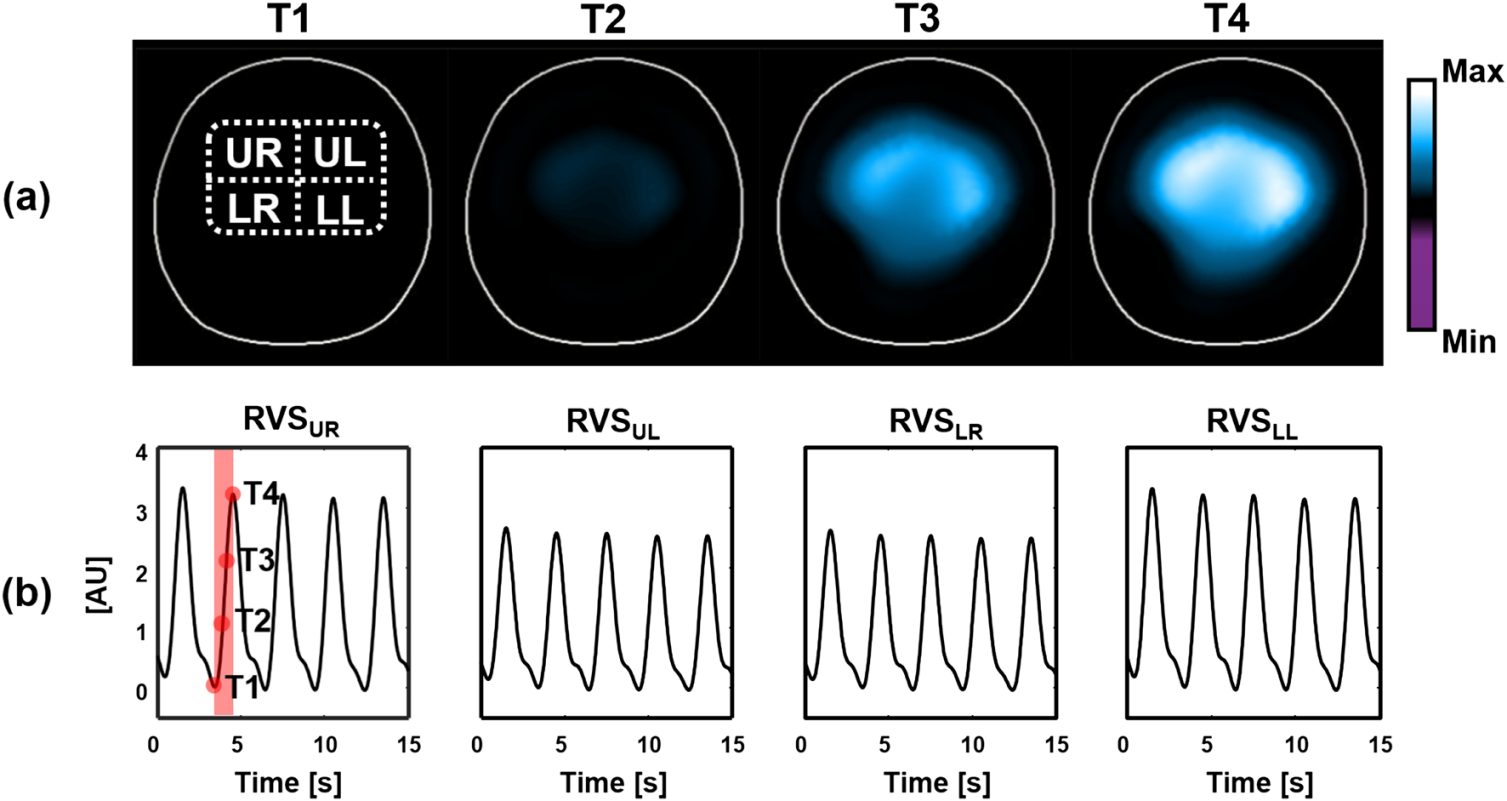
(**a**) Reconstructed images of lung ventilation and (**b**) extracted RVSs from four ROIs. The RVSs were periodic with little variability since the animal was mechanically ventilated.

**Figure 10:**
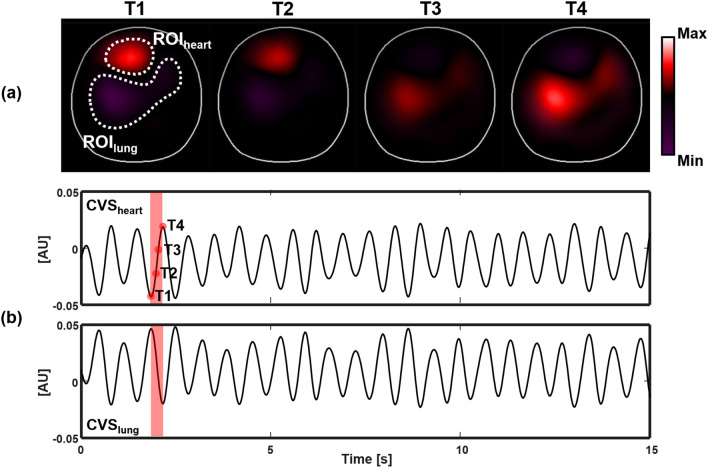
(**a**) Reconstructed images of cardiac blood flow and (**b**) extracted CVSs from two ROIs. Note that two CVSs from the heart and lung ROIs have the $180^\circ$ phase difference. The beat-to-beat variations of the CVSs are mainly due to changes in intrathoracic pressure during breathing cycles.

Figure 7 shows the synthesized data of 208 voltage channels for lung ventilation and magnified views of several channels. Figure 8 plots the synthesized voltage data for cardiac blood flow. Using these synthesized voltage channel data, time-difference EIT image reconstructions were conducted for lung ventilation and cardiac blood flow separately. Figure 9a,b show typical images of lung ventilation and extracted RVSs from four ROIs, respectively. Figure 10a,b show typical images of cardiac blood flow and extracted CVSs from two ROIs, respectively.

**Figure 11:**
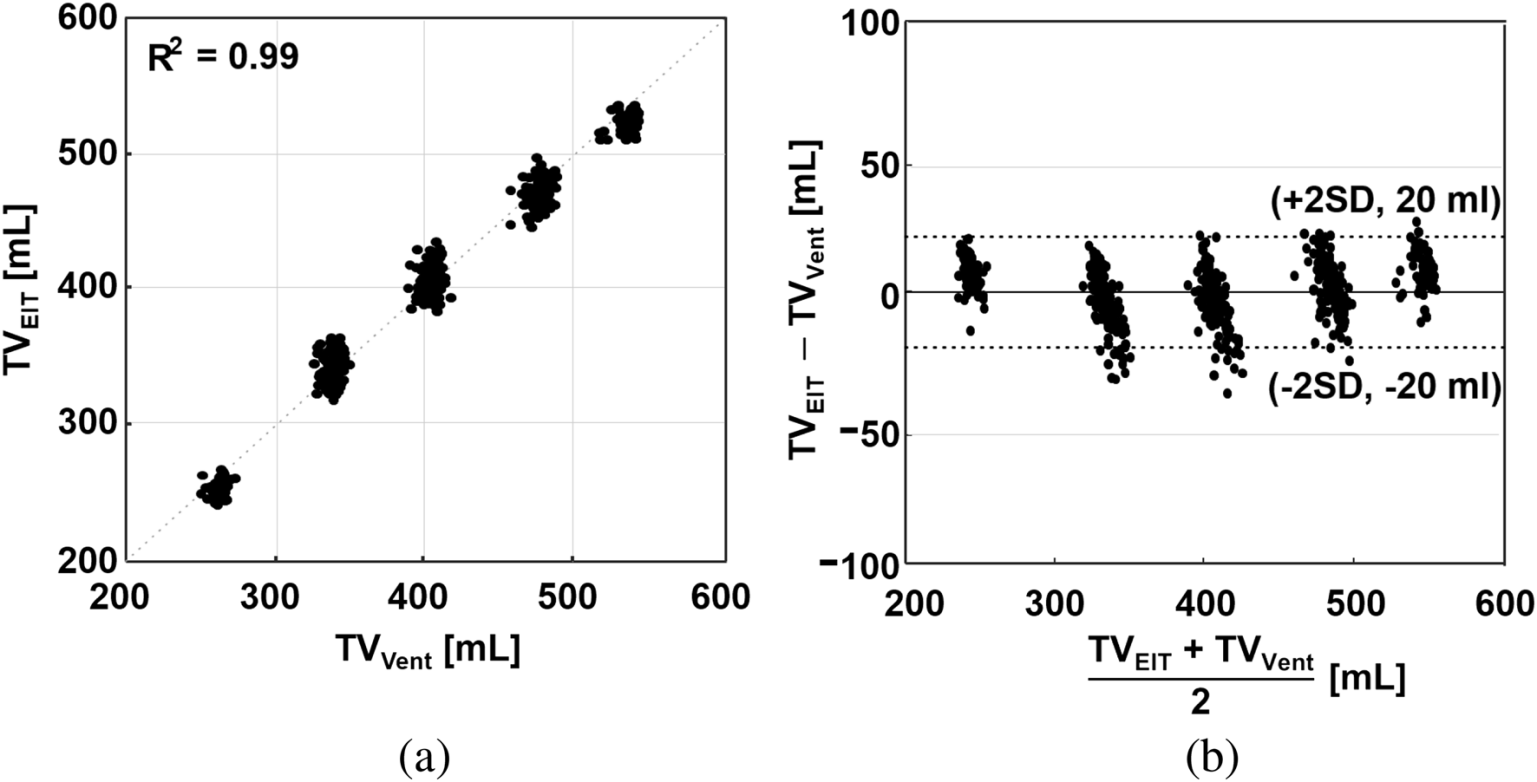
(**a**) Linear relation between the EIT-derived tidal volume ($TV_{EIT}$) and the supplied air volume from the mechanical ventilator ($TV_{Vent}$) with $R^2=0.99$. (**b**) Bland–Altman plot between them with ± 20 ml differences.

The total RVS was calibrated with the corresponding air volume from the mechanical ventilator. Tidal volumes were computed as valley-to-peak values of the total RVS. Using the data from six animals, the estimated $R^2$ value was 0.99 between the EIT-derived tidal volumes and the supplied air volumes from the mechanical ventilator as shown in Fig. 11a. The Bland–Altman plot shown in Fig. 11b indicates that the differences between them are less than ± 20 ml.

**Figure 12:**
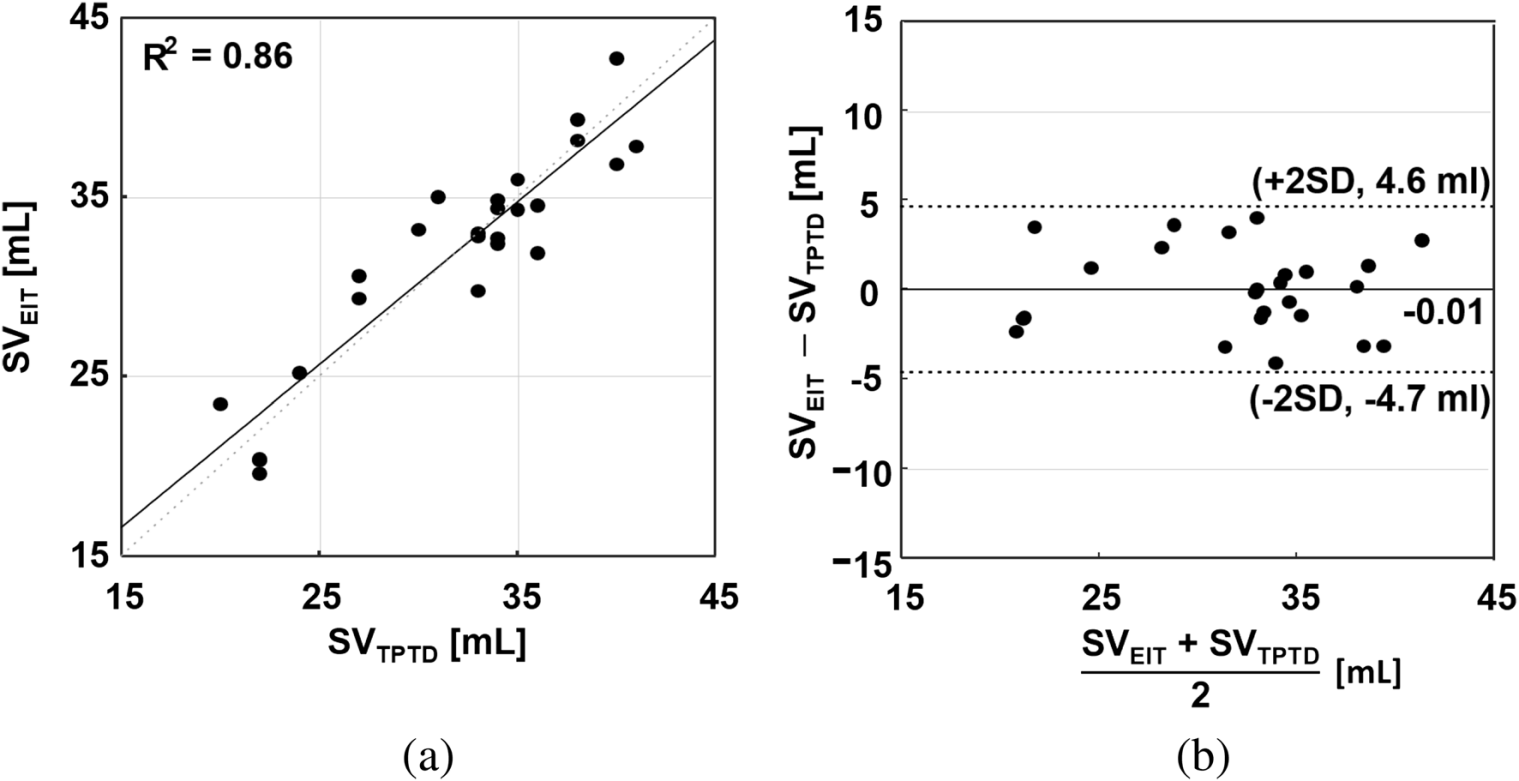
(**a**) Linear relation between the EIT-derived stroke volume ($SV_{EIT}$) and the simultaneously measured stroke volume using the TPTD method ($SV_{TPTD}$) with $R^2=0.86$. (**b**) Bland–Altman plot between them with ± 4.7 ml differences. There was no intervention to control the stroke volume other than controlling the tidal volume using the mechanical ventilator.

The CVS from the heart ROI was calibrated with the separately measured stroke volume data using the invasive hemodynamic monitor in the TPTD mode. Stroke volumes were computed as valley-to-peak values of the CVS from the heart ROI. The estimated $R^2$ value was 0.86 between the EIT-derived stroke volumes and the simultaneously measured stroke volumes using the TPTD method as shown in Fig. 12a. The Bland–Altman plot shown in Fig. 12b indicates that the differences between them are less than ± 4.7 ml.

**Figure 13:**
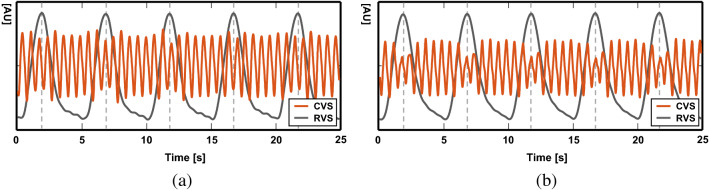
Stroke volume variation (SVV) was observed in the measured CVS during multiple breathing cycles at (**a**) normovolemia and (**b**) hypovolemia caused by bleeding. The average SVV during four breathing cycles was 31 and 59% for (**a**) and (**b**), respectively.

Stroke volume changes during a breathing cycle since intrathoracic pressure varies as lung volume changes. Figure 13a,b show two examples of the RVS and CVS at normovolemia and hypovolemia caused by bleeding, respectively. The amount of stroke volume changes during a breathing cycle is called the stroke volume variation (SVV)^29,30^. We calculated the SVV for the CVSs in Fig. 13 as

11 $$\begin{aligned} SVV=\frac{SV_{max}-SV_{min}}{(SV_{max}+SV_{min})/2}\times 100 (\%). \end{aligned}$$

The average SVV during four breathing cycles at normovolemia in Fig. 13a and hypovolemia in Fig. 13b was 31 and 59%, respectively.

## Discussion

We proposed a new method of using the PCA and ICA to extract shape-reference waveforms of lung ventilation and cardiac blood flow from measured boundary voltage data in EIT. Applying the source consistency method, all voltage channels associated with lung ventilation and cardiac blood flow could be separately synthesized using the corresponding extracted shape-reference waveforms. The data synthesis method improves the SNR of the voltage channel data. This is especially useful for cardiac blood flow imaging since the boundary voltages originating from cardiac blood flow are much smaller than those of lung ventilation. The source consistency method accomplishes this at the price of forcing the timecources of all pixel values within one source to have the same shape but possibly different amplitudes^22^. In a reconstructed EIT image using the source consistency method, therefore, we cannot recover different time delays among pixels.

Although the source consistency method imposes this constraint in the time domain, image reconstructions are done using a conventional sensitivity matrix method. Unlike the monotonicity assumption^31^, therefore, some pixel values can be increasing while others are decreasing. This means that the proposed method can recover a spatially varying conductivity distribution associated with each source. In Fig. 9b, all four regional RVSs have the same shape as the shape-reference waveform of lung ventilation in Fig. 6b, but they have different amplitudes. In Fig. 10b, two regional CVSs from the heart and lung ROIs have the same shape as the shape-reference waveform of cardiac blood flow in Fig. 6c, but they are 180$^\circ$ out of phase.

If the extracted shape-reference waveforms are the true representatives of lung ventilation and cardiac blood flow, the proposed method should effectively suppress all artifacts not associated with these physiological functions. The shape-reference waveforms, however, could have been affected by movements of internal organs and chest wall. The heart, for example, moves inside the chest due to respiration and its own beating. The geometrical relation between the electrodes and the heart, therefore, changes with time. Although the effective slice thickness of the reconstructed EIT images using one ring of 16 electrodes around the chest could be as large as 10 cm^9^, it needs to be investigated if the accuracy in measured stroke volume can be improved using a three-dimensional electrode configuration. Two rings of electrodes around the chest or only on the chest could be tried in future studies.

To extract shape-reference waveforms of lung ventilation and cardiac blood flow using the PCA and ICA methods, there should be a scout imaging period of one minute, for example, from a stationary subject with normal breathing. The heart rate should be measured independently from the subject using simultaneously acquired ECG or photoplethysmography data. The extracted shape-reference waveforms could be used for subsequent EIT imaging of lung ventilation and cardiac blood flow as long as these time-varying physiological functions remain reasonably stationary. In this paper, we tested the proposed method on mechanically ventilated animals with normal cardiac functions. In practice, however, spontaneous breathing could be irregular and there may occur cardiac arrhythmias such as atrial fibrillation. To be able to properly handle more complicated patterns of lung ventilation and cardiac blood flow, it will be worthwhile to carefully investigate the information contents of multiple PCA and ICA components to check the possibility of using a sum of multiple components as a shape-reference waveform. Future studies should also improve the real-time implementation of the proposed method to determine when and how to update the shape-reference waveforms based on a quality estimate of reconstructed images.

The proposed method may work well in the absence of excessive motion artifacts. Once the shape-reference waveforms are extracted, the method can produce the RVS and CVS even though some motion artifacts occur afterwards. In practice, however, there could be different amounts of motion artifacts from patients in ICU, PACU, ER, and other places in hospital. Further studies are needed to find how robust is the method against motion artifacts from patients in numerous clinical settings.

All impedance-based methods including EIT to measure air or blood volumes inside the human body provide values of impedance or impedance changes not absolute volumes in milliliter. The RVS and CVS extracted from EIT images could be used without any volume calibration for some clinical applications where only changes in volume over time are the key information. However, there are other clinical applications requesting absolute values of stroke volume and tidal volume. Future studies should devise a volume calibration method using personal information such as age, sex, weight, and height in addition to measured impedance data from each subject.

Among many potential clinical applications, the RVS could be used to monitor hypoventilation of non-intubated patients with respiratory depression^32^. The CVS could contribute to optimize the fluid management protocol for hemodynamically-unstable patients in ICU^33^. Future studies are needed to show the clinical efficacy of the proposed method in these clinical applications. Other clinical applications of noninvasive cardiopulmonary monitoring in numerous clinical settings could be also pursued.

## Conclusion

We developed a new noninvasive method to simultaneously measure stroke volume and tidal volume using EIT with the PCA, ICA, and source consistency method. The measured stroke volume and tidal volume values from six anesthetized animals were compared with those simultaneously measured using the invasive transpulmonary thermodilution method and the mechanical ventilator, respectively. The differences in stroke volume were within ± 4.7 ml in the range of 15–45 ml. The differences in tidal volume were within ± 20 ml in the range of 200 and 600 ml. Future studies are needed to further validate the measurement accuracy in wider ranges of stroke volume and tidal volume. Clinical studies should be pursued to find the efficacy of the proposed method in applications such as fluid management, administration of vasopressor and inotrope, hypoventilation detection, sleep study, and others.

## Data Availability

The data that support the findings of this study are available from the corresponding author, [EJW], upon reasonable request.

## References

[1] Henderson RP, Webster JG (1978). An impedance camera for spatially specific measurements of the thorax. IEEE Trans. Biomed. Eng..

[2] Metherall P, Barber DC, Smallwood RH, Brown BH (1996). Three-dimensional electrical impedance tomography. Nature.

[3] Cheney M, Isaacson D, Newell JC (1999). Electrical impedance tomography. SIAM Rev..

[4] Holder DS (2005). Electrical Impedance Tomography: Methods, History and Applications.

[5] Gabriel C, Gabriel S, Corthout E (1996). The dielectric properties of biological tissues: I. Literature survey. Phys. Med. Biol..

[6] Malmivuo J, Plonsey R (1995). Bioelectromagnetism: Principles and Applications of Bioelectric and Biomagnetic Fields.

[7] Seo JK, Woo EJ (2013). Nonlinear Inverse Problems in Imaging.

[8] Frerichs I, Amato MB, Van Kaam AH, Tingay DG, Zhao Z, Grychtol B, Bodenstein M, Gagnon H, Böhm SH, Teschner E, Stenqvist O, Mauri T, Torsani V, Camporota L, Schibler A, Wolf GK, Gommers D, Leonhardt S, Adler A (2017). Chest electrical impedance tomography examination, data analysis, terminology, clinical use and recommendations: consensus statement of the TRanslational EIT developmeNt stuDy group. Thorax.

[9] Adler A, Boyle A (2017). Electrical impedance tomography: tissue properties and image measures. IEEE Trans. Biomed. Eng..

[10] Brown BH, Leathard A, Sinton A, McArdle FJ, Smith RWM, Barber DC (1992). Blood flow imaging using electrical impedance tomography. Clin. Phys. Physiol. Meas..

[11] Kerrouche N, McLeod CN, Lionheart WRB (2001). Time series of EIT chest images using singular value decomposition and Fourier transform. Physiol. Meas..

[12] Zlochiver S, Freimark D, Arad M, Adunsky A, Abboud S (2006). Parametric EIT for monitoring cardiac stroke volume. Physiol. Meas..

[13] Deibele JM, Luepschen H, Leonhardt SL (2008). Dynamic separation of pulminary and cardiac changes in electrical impedance tomography. Physiol. Meas..

[14] Pikkemaat R, Lundin S, Stenqvist O, Hilgers RD, Leonhardt S (2014). Recent advances in and limitations of cardiac output monitoring by means of electrical impedance tomography. Anesth. Analg..

[15] Rahman T, Hasan MM, Farooq A, Uddin MD (2013). Extraction of cardiac and respiration signals in electrical impedance tomography based on independent component analysis. J. Elec. Bioimp..

[16] Frerichs I, Hinz J, Herrmann P, Weisser G, Hahn G, Quintel M, Hellige G (2002). Regional lung perfusion as determined by electrical impedance tomography in comparison with electron beam CT imaging. IEEE Trans. Med. Imag..

[17] Frerichs I, Pulletz S, Elke G, Reifferscheid F, Schadler D, Scholz J, Weiler N (2009). Assessment of changes in distribution of lung perfusion by electrical impedance tomography. Resp..

[18] Borges JB, Suarez-Sipmann F, Bohm SH, Tusman G, Melo A, Maripuu E, Sandstrom M, Park M, Costa ELV, Hedenstierna G, Amato M (2012). Regional lung perfusion estimated by electrical impedance tomography in a piglet model of lung collapse. J. Appl. Physiol..

[19] Leonhardt S, Lachmann B (2012). Electrical impedance tomography: the holy grail of ventilation and perfusion monitoring?. Int. Care Med..

[20] M. Proenca *Noninvasive hemodynamic monitoring by electrical impedance tomography*. PhD Thesis, EPFL, Lausanne, Switzerland (2017).

[21] F. Braun *Noninvasive Stroke Volume Monitoring by Electrical Impedance Tomography*. PhD Thesis, EPFL, Lausanne, Switzerland (2018).

[22] Zhang T, Jang GY, Oh TI, Jeung KW, Wi H, Woo EJ (2020). Source consistency electrical impedance tomography. SIAM Appl. Math..

[23] Oh TI, Woo EJ, Holder D (2007). Multi-frequency EIT system with radially symmetric architecture: KHU Mark1. Physiol. Meas..

[24] Oh TI, Lee KH, Kim SM, Koo W, Woo EJ, Holder D (2007). Calibration methods for a multi-channel multi-frequency EIT system. Physiol. Meas..

[25] Oh TI, Koo W, Lee KH, Kim SM, Lee J, Kim SW, Seo JK, Woo EJ (2008). Validation of a multi-frequency electrical impedance tomography (mfEIT) system KHU Mark1: impedance spectroscopy and time-difference imaging. Physiol. Meas..

[26] Oh TI, Wi H, Kim DY, Yoo PJ, Woo EJ (2011). A fully parallel multi-frequency EIT system with flexible electrode configuration: KHU Mark2. Physiol. Meas..

[27] Wi H, Sohal H, McEwan AL, Woo EJ, Oh TI (2014). Multi-frequency electrical impedance tomography system with automatic self-calibration for long-term monitoring. IEEE Trans. Biomed. Cir. Sys..

[28] Lee K, Woo EJ, Seo JK (2017). A fidelity-embedded regularization method for robust electrical impedance tomography. IEEE Trans. Med. Imag..

[29] Berkenstadt H, Margalit N, Hadani M, Friedman Z, Segal E, Villa Y, Perel A (2001). Stroke volume variation as a predictor of fluid responsiveness in patients undergoing brain surgery. Anesth. Analg..

[30] Benes J, Chytra I, Altmann P, Hluchy M, Kasal E, Svitak R, Pradl R, Stepan M (2010). Intraoperative fluid optimization using stroke volume variation in high risk surgical patients: Results of prospective randomized study. Crit. Care.

[31] Zhou L, Harrach B, Seo JK (2018). Monotonicity-based electrical impedance tomography for lung imaging. Inv. Prob..

[32] R. M. Gupta and D. A. Edwards, Monitoring for opiod-induced respiratory depression, *APSF Newsl.*, Feb., pp 70–72 (2018).

[33] Cecconi M, Hofer C, Teboul JL, Pettila V, Wilkman E, Molnar Z, Della Rocca G, Aldecoa C, Artigas A, Jog S, Sander M, Spies C, Lefrant JY, Be Backer D (2015). Fluid challenges in intensive care: the FENICE study. Int. Care Med..

